# 2,2′-Azanediyl­diethanaminium pyridine-2,5-dicarboxyl­ate

**DOI:** 10.1107/S1600536810054413

**Published:** 2011-01-08

**Authors:** Hossein Aghabozorg, Maryam Saemi, Zeynab Khazaei, Vahid Amani, Behrouz Notash

**Affiliations:** a Faculty of Chemistry, Tarbiat Moallem University, 15614 Tehran, Iran; b Department of Chemistry, Shahid Beheshti University, G.C., Evin, Tehran 1983963113, Iran

## Abstract

The crystal structure of the title compound, C_4_H_15_N_3_^2+^·C_7_H_3_NO_4_^2−^, consists of diethyl­enetriaminium (2,2′-azanediyl­diethanaminium) cations and pyridine-2,5-dicarboxyl­ate anions, which are linked by N—H⋯O, N—H⋯N and C—H⋯O hydrogen bonds. C—H⋯π inter­actions are also observed. In the anion, the carboxyl­ate groups are oriented at dihedral angles of 11.04 (15) and 6.31 (14)° with respect to the pyridine ring.

## Related literature

For general background to proton-transfer compounds, see: Sheshmani *et al.* (2007 ▶); Aghabozorg *et al.* (2008*a* ▶,*b* ▶,*c* ▶); Derikvand *et al.* (2009 ▶).
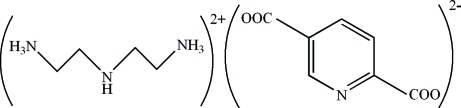


## Experimental

### 

#### Crystal data

C_4_H_15_N_3_^2+^·C_7_H_3_NO_4_^2−^
*M* *_r_* = 270.29
Monoclinic, 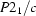

*a* = 10.485 (2) Å
*b* = 7.7016 (15) Å
*c* = 17.254 (4) Å
β = 106.67 (3)°
*V* = 1334.7 (5) Å^3^
*Z* = 4
Mo *K*α radiation
μ = 0.10 mm^−1^
*T* = 298 K
0.3 × 0.3 × 0.15 mm

#### Data collection

Stoe IPDS II diffractometer
Absorption correction: integration (*X-RED32*; Stoe & Cie, 2005 ▶) *T*_min_ = 0.967, *T*_max_ = 0.983
14267 measured reflections
3593 independent reflections
2523 reflections with *I* > 2σ(*I*)
*R*_int_ = 0.099

#### Refinement

*R*[*F* ^2^ > 2σ(*F* ^2^)] = 0.087
*wR*(*F* ^2^) = 0.184
*S* = 1.18
3593 reflections
200 parameters
H atoms treated by a mixture of independent and constrained refinement
Δρ_max_ = 0.39 e Å^−3^
Δρ_min_ = −0.28 e Å^−3^

### 

Data collection: *X-AREA* (Stoe & Cie, 2005 ▶); cell refinement: *X-AREA*; data reduction: *X-RED32* (Stoe & Cie, 2005 ▶); program(s) used to solve structure: *SHELXS97* (Sheldrick, 2008 ▶); program(s) used to refine structure: *SHELXL97* (Sheldrick, 2008 ▶); molecular graphics: *ORTEP-3 for Windows* (Farrugia, 1997 ▶); software used to prepare material for publication: *WinGX* (Farrugia, 1999 ▶).

## Supplementary Material

Crystal structure: contains datablocks I, global. DOI: 10.1107/S1600536810054413/xu5129sup1.cif

Structure factors: contains datablocks I. DOI: 10.1107/S1600536810054413/xu5129Isup2.hkl

Additional supplementary materials:  crystallographic information; 3D view; checkCIF report

## Figures and Tables

**Table 1:** Hydrogen-bond geometry (Å, °) *Cg* is the centroid of the pyridine ring.

*D*—H⋯*A*	*D*—H	H⋯*A*	*D*⋯*A*	*D*—H⋯*A*
N2—H2*A*⋯O3^i^	0.96 (3)	2.56 (3)	3.254 (3)	130 (2)
N2—H2*A*⋯O4^i^	0.96 (3)	1.94 (3)	2.886 (3)	169 (3)
N2—H2*B*⋯O1	0.90 (3)	1.98 (3)	2.821 (4)	155 (3)
N2—H2*C*⋯O3^ii^	0.99 (4)	1.87 (4)	2.843 (3)	167 (3)
N3—H3*A*⋯O2^iii^	0.97 (4)	2.38 (4)	3.223 (3)	145 (3)
N4—H4*A*⋯O2	0.95 (4)	1.91 (4)	2.807 (4)	158 (3)
N4—H4*B*⋯O4^ii^	0.94 (4)	1.92 (3)	2.840 (3)	167 (4)
N4—H4*C*⋯O2^iv^	0.91 (4)	1.98 (4)	2.823 (4)	154 (4)
N4—H4*C*⋯N1^iv^	0.91 (4)	2.57 (4)	3.253 (4)	133 (3)
C8—H8*A*⋯O1^v^	0.97	2.49	3.218 (4)	132
C3—H3⋯*Cg*^i^	0.93	2.83	3.588 (3)	139
C10—H10*B*⋯*Cg*^iii^	0.97	2.91	3.846 (3)	161

Symmetry codes: (i) 
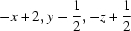
; (ii) 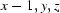; (iii) 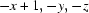; (iv) 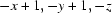; (v) 
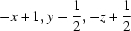
.

**Fig. 1.**
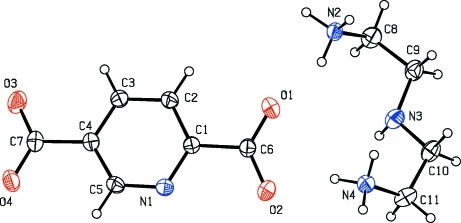
The molecular structure of the title molecule, with the atom-numbering scheme. Displacement ellipsoids are drawn at the 50% probability level.

**Fig. 2.**
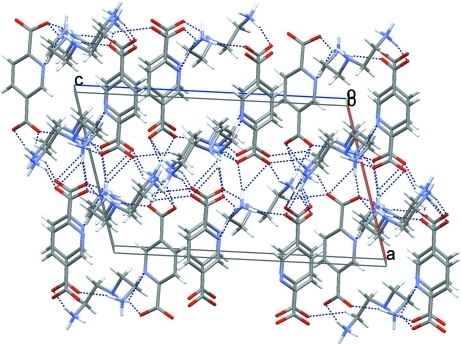
The unit-cell packing diagram for the title molecule.

**Fig. 3.**
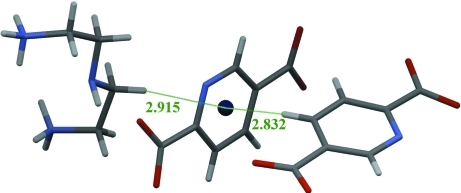
Intermolecular C—H···π interactions for the title molecule.

## supplementary crystallographic information

### Comment

Proton transfer is very important in physics, chemistry and biochemistry. In order to develop new types of proton transfer compounds and hydrogen bonding systems, our research group has already synthesized proton transfer compounds with different proton donors and acceptors (Sheshmani *et al.*, 2007; Aghabozorg *et al.*, 2008*a*; Aghabozorg *et al.*, 2008*b*; Aghabozorg *et al.*, 2008*c*; Derikvand *et al.*, 2009). We herein report the crystal structure of the title compound.

The molecular structure of the title compound is shown in Fig. 1. The crystal structure shows that two protons from two carboxylic acid groups are transferred to two N atoms of the diethylenetriamine.

As can be seen from the packing diagram (Fig. 2), there are variety intera and intermolecular N—H···O, N—H···N and C—H···O hydrogen bonds (Table 1) in the crystal structure

Also, as shown in Fig. 3, there are C—H···π interactions between C10—H10B bond of diethylenetriaminium ion and pyridine ring and C3—H3 bond of pyridine-2,5-dicarboxylate ion and symmetry-related pyridine ring in the crystal structure [distance from centroid = 2.91 and 2.83 Å; angle = 161 and 139 ° and symmetry codes: 1 - *x*, -*y*, -*z* and 2 - *x*, -1/2 + *y*, 1/2 - *z*, respectively].

Intermolecular N—H···O, N—H···N and C—H···O hydrogen bonds and C—H···π interactions in this compound seem to be effective in the stabilization of the crystal structure, resulting in the formation of a three-dimensional supramolecular structure.

### Experimental

Diethylenetriamine (0.28 g, 0.29 ml, 2.66 mmol) was added to a solution of pyridine-2,5-dicarboxylic acid (0.45 g, 2.66 mmol) in methanol (50 ml) at room temperature. The suitable crystals for X-ray diffraction experiment were obtained by methanol diffusion to a pale yellow solution in water. Suitable crystals were isolated after one week (yield; 0.55 g, 76.5%).

### Refinement

H atoms bonded to N atoms were located in a difference Fourier map and refined isotropically. Other H atoms were positioned geometrically with C—H = 0.93 Å for aromatic and 0.97 Å for methylene, and constrained to ride on their parent atoms with U_iso_(H) = 1.2U_eq_(C).

### Crystal data

**Table:**

C_4_H_15_N_3_^2+^·C_7_H_3_NO_4_^2^^−^	*F*(000) = 576.0
*M**_r_* = 270.29	*D*_x_ = 1.345 Mg m^−^^3^
Monoclinic, *P*2_1_/*c*	Mo *K*α radiation, λ = 0.71073 Å
Hall symbol: -P 2ybc	Cell parameters from 3595 reflections
*a* = 10.485 (2) Å	θ = 2.5–29.2°
*b* = 7.7016 (15) Å	µ = 0.10 mm^−^^1^
*c* = 17.254 (4) Å	*T* = 298 K
β = 106.67 (3)°	Block, yellow
*V* = 1334.7 (5) Å^3^	0.3 × 0.3 × 0.15 mm
*Z* = 4	

### Data collection

**Table:**

Stoe IPDS II diffractometer	3593 independent reflections
Radiation source: fine-focus sealed tube	2523 reflections with *I* > 2σ(*I*)
graphite	*R*_int_ = 0.099
Detector resolution: 0.15 mm pixels mm^-1^	θ_max_ = 29.2°, θ_min_ = 2.5°
rotation method scans	*h* = −14→13
Absorption correction: integration (*X-RED32*; Stoe & Cie, 2005)	*k* = −10→10
*T*_min_ = 0.967, *T*_max_ = 0.983	*l* = −23→23
14267 measured reflections	

### Refinement

**Table:**

Refinement on *F*^2^	Primary atom site location: structure-invariant direct methods
Least-squares matrix: full	Secondary atom site location: difference Fourier map
*R*[*F*^2^ > 2σ(*F*^2^)] = 0.087	Hydrogen site location: inferred from neighbouring sites
*wR*(*F*^2^) = 0.184	H atoms treated by a mixture of independent and constrained refinement
*S* = 1.18	*w* = 1/[σ^2^(*F*_o_^2^) + (0.0436*P*)^2^ + 0.9032*P*] where *P* = (*F*_o_^2^ + 2*F*_c_^2^)/3
3593 reflections	(Δ/σ)_max_ < 0.001
200 parameters	Δρ_max_ = 0.39 e Å^−^^3^
0 restraints	Δρ_min_ = −0.28 e Å^−^^3^

### Special details

**Table:**

Geometry. All e.s.d.'s (except the e.s.d. in the dihedral angle between two l.s. planes) are estimated using the full covariance matrix. The cell e.s.d.'s are taken into account individually in the estimation of e.s.d.'s in distances, angles and torsion angles; correlations between e.s.d.'s in cell parameters are only used when they are defined by crystal symmetry. An approximate (isotropic) treatment of cell e.s.d.'s is used for estimating e.s.d.'s involving l.s. planes.
Refinement. Refinement of *F*^2^ against ALL reflections. The weighted *R*-factor *wR* and goodness of fit *S* are based on *F*^2^, conventional *R*-factors *R* are based on *F*, with *F* set to zero for negative *F*^2^. The threshold expression of *F*^2^ > σ(*F*^2^) is used only for calculating *R*-factors(gt) *etc*. and is not relevant to the choice of reflections for refinement. *R*-factors based on *F*^2^ are statistically about twice as large as those based on *F*, and *R*- factors based on ALL data will be even larger.

### Fractional atomic coordinates and isotropic or equivalent isotropic displacement parameters (Å^2^)

**Table:**

	*x*	*y*	*z*	*U*_iso_*/*U*_eq_	
O1	0.6009 (2)	0.1955 (4)	0.15541 (15)	0.0729 (8)	
O2	0.60280 (19)	0.3262 (3)	0.04174 (12)	0.0548 (6)	
O3	1.29302 (19)	0.1713 (3)	0.26621 (13)	0.0551 (6)	
O4	1.28741 (19)	0.3736 (3)	0.17248 (12)	0.0509 (5)	
N1	0.8735 (2)	0.3611 (3)	0.09555 (13)	0.0391 (5)	
N2	0.4579 (2)	−0.0627 (3)	0.21118 (15)	0.0382 (5)	
N3	0.2987 (2)	−0.0099 (3)	0.04917 (15)	0.0460 (6)	
N4	0.3271 (3)	0.3560 (4)	0.01663 (16)	0.0443 (6)	
C1	0.8097 (2)	0.2669 (3)	0.13871 (15)	0.0327 (5)	
C2	0.8779 (3)	0.1723 (4)	0.20632 (17)	0.0436 (6)	
H2	0.8315	0.1112	0.2361	0.052*	
C3	1.0150 (3)	0.1698 (4)	0.22901 (16)	0.0422 (6)	
H3	1.0619	0.1033	0.2729	0.051*	
C4	1.0825 (2)	0.2672 (3)	0.18589 (15)	0.0341 (5)	
C5	1.0069 (3)	0.3606 (3)	0.12018 (16)	0.0381 (6)	
H5	1.0515	0.4273	0.0913	0.046*	
C6	0.6586 (2)	0.2626 (3)	0.10991 (16)	0.0381 (6)	
C7	1.2334 (3)	0.2704 (4)	0.21038 (16)	0.0401 (6)	
C8	0.3824 (3)	−0.2064 (4)	0.16064 (19)	0.0496 (7)	
H8A	0.3570	−0.2911	0.1951	0.059*	
H8B	0.4386	−0.2640	0.1326	0.059*	
C9	0.2599 (3)	−0.1373 (4)	0.09994 (18)	0.0475 (7)	
H9A	0.2120	−0.2316	0.0669	0.057*	
H9B	0.2015	−0.0841	0.1277	0.057*	
C10	0.1898 (3)	0.0876 (4)	−0.00271 (17)	0.0474 (7)	
H10A	0.1346	0.1334	0.0290	0.057*	
H10B	0.1353	0.0113	−0.0437	0.057*	
C11	0.2408 (3)	0.2359 (4)	−0.04313 (17)	0.0525 (8)	
H11A	0.2909	0.1892	−0.0777	0.063*	
H11B	0.1657	0.3001	−0.0770	0.063*	
H2A	0.539 (3)	−0.100 (4)	0.249 (2)	0.058 (9)*	
H2B	0.486 (3)	0.011 (4)	0.1790 (18)	0.044 (8)*	
H2C	0.405 (4)	0.010 (5)	0.238 (2)	0.069 (10)*	
H3A	0.352 (4)	−0.064 (5)	0.018 (2)	0.083 (12)*	
H4A	0.417 (4)	0.320 (4)	0.032 (2)	0.059 (9)*	
H4B	0.300 (4)	0.365 (5)	0.064 (2)	0.068 (11)*	
H4C	0.322 (4)	0.463 (5)	−0.006 (2)	0.078 (12)*	

### Atomic displacement parameters (Å^2^)

**Table:**

	*U*^11^	*U*^22^	*U*^33^	*U*^12^	*U*^13^	*U*^23^
O1	0.0440 (12)	0.103 (2)	0.0698 (15)	−0.0193 (12)	0.0137 (11)	0.0258 (14)
O2	0.0361 (10)	0.0691 (14)	0.0538 (12)	0.0065 (10)	0.0041 (9)	0.0145 (11)
O3	0.0376 (10)	0.0646 (14)	0.0590 (13)	0.0127 (10)	0.0074 (9)	0.0041 (11)
O4	0.0381 (10)	0.0631 (13)	0.0544 (12)	−0.0137 (9)	0.0176 (9)	−0.0085 (10)
N1	0.0391 (11)	0.0379 (11)	0.0397 (11)	−0.0011 (9)	0.0103 (9)	0.0061 (9)
N2	0.0306 (11)	0.0380 (12)	0.0428 (12)	−0.0004 (9)	0.0055 (10)	0.0057 (10)
N3	0.0443 (13)	0.0444 (13)	0.0448 (13)	0.0052 (10)	0.0055 (10)	0.0025 (11)
N4	0.0377 (12)	0.0476 (14)	0.0454 (14)	0.0005 (11)	0.0084 (10)	0.0097 (11)
C1	0.0326 (11)	0.0284 (11)	0.0369 (12)	0.0018 (9)	0.0096 (10)	0.0012 (10)
C2	0.0364 (13)	0.0458 (15)	0.0480 (15)	−0.0022 (11)	0.0108 (11)	0.0151 (12)
C3	0.0366 (13)	0.0429 (14)	0.0450 (14)	0.0023 (11)	0.0083 (11)	0.0119 (12)
C4	0.0336 (12)	0.0303 (12)	0.0387 (13)	−0.0013 (10)	0.0108 (10)	−0.0055 (10)
C6	0.0323 (12)	0.0391 (13)	0.0414 (14)	−0.0006 (10)	0.0084 (10)	0.0004 (11)
C7	0.0333 (12)	0.0418 (14)	0.0446 (14)	−0.0002 (11)	0.0105 (11)	−0.0114 (12)
C8	0.0522 (17)	0.0348 (14)	0.0556 (17)	−0.0015 (12)	0.0056 (14)	0.0031 (12)
C9	0.0450 (15)	0.0411 (14)	0.0493 (15)	−0.0075 (12)	0.0021 (12)	−0.0018 (13)
C10	0.0462 (15)	0.0427 (15)	0.0451 (15)	0.0017 (12)	0.0000 (12)	−0.0072 (12)
C11	0.0604 (18)	0.0531 (17)	0.0379 (14)	0.0071 (15)	0.0040 (13)	0.0039 (13)
C5	0.0414 (13)	0.0343 (12)	0.0403 (13)	−0.0041 (11)	0.0146 (11)	0.0039 (11)

### Geometric parameters (Å, °)

**Table:**

O1—C6	1.233 (3)	C1—C6	1.519 (3)
O2—C6	1.253 (3)	C2—C3	1.378 (4)
O3—C7	1.248 (3)	C2—H2	0.9300
O4—C7	1.262 (3)	C3—C4	1.385 (4)
N1—C5	1.340 (3)	C3—H3	0.9300
N1—C1	1.347 (3)	C4—C5	1.384 (4)
N2—C8	1.489 (4)	C4—C7	1.516 (3)
N2—H2A	0.96 (3)	C8—C9	1.503 (4)
N2—H2B	0.90 (3)	C8—H8A	0.9700
N2—H2C	1.00 (4)	C8—H8B	0.9700
N3—C10	1.444 (4)	C9—H9A	0.9700
N3—C9	1.450 (4)	C9—H9B	0.9700
N3—H3A	0.98 (4)	C10—C11	1.514 (4)
N4—C11	1.484 (4)	C10—H10A	0.9700
N4—H4A	0.94 (4)	C10—H10B	0.9700
N4—H4B	0.95 (4)	C11—H11A	0.9700
N4—H4C	0.91 (4)	C11—H11B	0.9700
C1—C2	1.387 (3)	C5—H5	0.9300
			
C5—N1—C1	117.5 (2)	O3—C7—O4	125.9 (2)
C8—N2—H2A	113.6 (19)	O3—C7—C4	117.2 (2)
C8—N2—H2B	108.7 (19)	O4—C7—C4	117.0 (2)
H2A—N2—H2B	103 (3)	N2—C8—C9	110.5 (2)
C8—N2—H2C	115 (2)	N2—C8—H8A	109.6
H2A—N2—H2C	111 (3)	C9—C8—H8A	109.6
H2B—N2—H2C	105 (3)	N2—C8—H8B	109.6
C10—N3—C9	114.7 (2)	C9—C8—H8B	109.6
C10—N3—H3A	111 (2)	H8A—C8—H8B	108.1
C9—N3—H3A	111 (2)	N3—C9—C8	109.2 (2)
C11—N4—H4A	112 (2)	N3—C9—H9A	109.8
C11—N4—H4B	112 (2)	C8—C9—H9A	109.8
H4A—N4—H4B	107 (3)	N3—C9—H9B	109.8
C11—N4—H4C	108 (2)	C8—C9—H9B	109.8
H4A—N4—H4C	109 (3)	H9A—C9—H9B	108.3
H4B—N4—H4C	108 (3)	N3—C10—C11	110.9 (2)
N1—C1—C2	122.0 (2)	N3—C10—H10A	109.5
N1—C1—C6	117.9 (2)	C11—C10—H10A	109.5
C2—C1—C6	120.1 (2)	N3—C10—H10B	109.5
C3—C2—C1	119.4 (2)	C11—C10—H10B	109.5
C3—C2—H2	120.3	H10A—C10—H10B	108.0
C1—C2—H2	120.3	N4—C11—C10	112.1 (2)
C2—C3—C4	119.5 (2)	N4—C11—H11A	109.2
C2—C3—H3	120.3	C10—C11—H11A	109.2
C4—C3—H3	120.3	N4—C11—H11B	109.2
C5—C4—C3	117.4 (2)	C10—C11—H11B	109.2
C5—C4—C7	121.8 (2)	H11A—C11—H11B	107.9
C3—C4—C7	120.8 (2)	N1—C5—C4	124.2 (2)
O1—C6—O2	125.4 (2)	N1—C5—H5	117.9
O1—C6—C1	117.2 (2)	C4—C5—H5	117.9
O2—C6—C1	117.4 (2)		
			
C5—N1—C1—C2	0.1 (4)	C5—C4—C7—O3	−174.3 (2)
C5—N1—C1—C6	−178.4 (2)	C3—C4—C7—O3	5.8 (4)
N1—C1—C2—C3	−2.0 (4)	C5—C4—C7—O4	5.8 (4)
C6—C1—C2—C3	176.4 (3)	C3—C4—C7—O4	−174.1 (2)
C1—C2—C3—C4	2.6 (4)	C10—N3—C9—C8	−170.1 (2)
C2—C3—C4—C5	−1.3 (4)	N2—C8—C9—N3	58.8 (3)
C2—C3—C4—C7	178.6 (3)	C9—N3—C10—C11	170.9 (2)
N1—C1—C6—O1	−170.7 (3)	N3—C10—C11—N4	−58.1 (3)
C2—C1—C6—O1	10.9 (4)	C1—N1—C5—C4	1.3 (4)
N1—C1—C6—O2	9.9 (4)	C3—C4—C5—N1	−0.7 (4)
C2—C1—C6—O2	−168.6 (3)	C7—C4—C5—N1	179.4 (2)

### Hydrogen-bond geometry (Å, °)

**Table:**

Cg is the centroid of the pyridine ring.

**Table:**

*D*—H···*A*	*D*—H	H···*A*	*D*···*A*	*D*—H···*A*
N2—H2A···O3^i^	0.96 (3)	2.56 (3)	3.254 (3)	130 (2)
N2—H2A···O4^i^	0.96 (3)	1.94 (3)	2.886 (3)	169 (3)
N2—H2B···O1	0.90 (3)	1.98 (3)	2.821 (4)	155 (3)
N2—H2C···O3^ii^	0.99 (4)	1.87 (4)	2.843 (3)	167 (3)
N3—H3A···O2^iii^	0.97 (4)	2.38 (4)	3.223 (3)	145 (3)
N4—H4A···O2	0.95 (4)	1.91 (4)	2.807 (4)	158 (3)
N4—H4B···O4^ii^	0.94 (4)	1.92 (3)	2.840 (3)	167 (4)
N4—H4C···O2^iv^	0.91 (4)	1.98 (4)	2.823 (4)	154 (4)
N4—H4C···N1^iv^	0.91 (4)	2.57 (4)	3.253 (4)	133 (3)
C8—H8A···O1^v^	0.97	2.49	3.218 (4)	132
C3—H3···Cg^i^	0.93	2.83	3.588 (3)	139
C10—H10B···Cg^iii^	0.97	2.91	3.846 (3)	161

Symmetry codes: (i) −*x*+2, *y*−1/2, −*z*+1/2; (ii) *x*−1, *y*, *z*; (iii) −*x*+1, −*y*, −*z*; (iv) −*x*+1, −*y*+1, −*z*; (v) −*x*+1, *y*−1/2, −*z*+1/2.
